# E-Learning to Fill Gaps in Serious Illness Communication Education: A Missing Piece or an Unfinished Puzzle?

**DOI:** 10.1089/pmr.2025.0004

**Published:** 2025-02-03

**Authors:** Justin J. Sanders

**Affiliations:** Department of Family Medicine, McGill University, Montreal, Canada.

Serious illness communication may be the key ingredient in the palliative care syringe. The benefits of palliative care are increasingly linked to the adaptive coping that results from clinicians’ cultivation of prognostic awareness, responsiveness to emotion, and support for decision-making and planning.^1,2^ Fittingly, these communication tasks comprise the majority of time spent by palliative care clinicians.^3^ The knowledge, attitudes, and skills that support these tasks—attention to setting, effective information transfer, and emotion—hold intuitive value for patients and caregivers but have received relatively scant attention from medical educators. That communication failures remain the key driver of dissatisfaction and complaints suggests that this remains one of the most important gaps in health care training at all levels.^4^

To some degree, the state of the science of serious illness communication parallels that of palliative care. We have moved from questions about whether it is the right thing to figuring out how to effectively deliver it. In the case of serious illness communication, this is a question about disseminating educational initiatives that transform institutional culture. That is, how do we scale teaching of the knowledge, attitudes, and skills in ways that help us cross the quality chasm that divides innovators and early adopters from everyone else. Over the past decade, leading U.S. and international health care institutions have attempted to do so by engaging programs such as VitalTalk and the Ariadne Lab’s Serious Illness Care Program to provide synchronous training to clinicians in unstructured and structured approaches to delivering bad news and goals of care and serious illness conversations.^5,6^ Some institutions have found great success in implementing these approaches,^7^ but many more have struggled to overcome challenges related to time and resources in the delivery of synchronous training and to the upkeep of skills beyond training Figure 1.

**FIG. 1.**
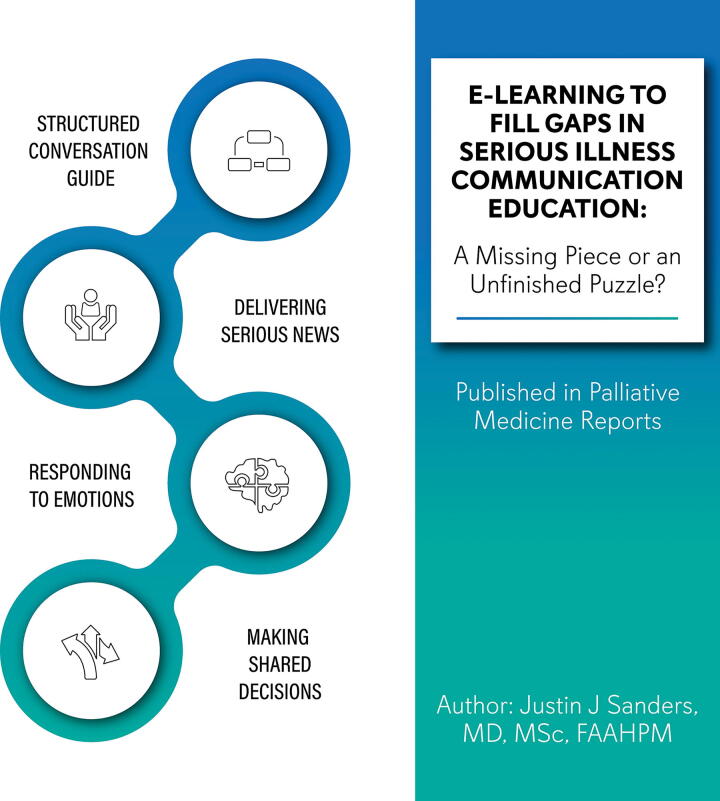
E-module learning for scaling serious illness communication skills teaching.

In an article in this issue of *Palliative Medicine Reports*, James and colleagues present findings from a pilot among family medicine and palliative care trainees of a serious illness communication-focused e-learning module: *E-module learning for scaling serious illness communication skills teaching: A pilot study in Family Medicine and Palliative Care,* https://doi.org/10.1089/pmr.2024.0048. One module focused on a structured conversation guide adapted from Ariadne Lab’s Serious Illness Conversation Guide and VitalTalk’s REMAP mnemonic. A second module focused on skills and approaches to delivering serious news, responding to emotion, and promoting shared decision-making. The authors note that these online modules can be completed independently and asynchronously. Learners were assessed using a survey of attitudes toward e-learning, generally, and the module, specifically, as well as a second survey assessing ongoing e-module use and confidence in leading serious illness conversations. In addition, family medicine and palliative care faculty were invited by their respective division heads to complete the e-modules and surveys focused on usability and relevance.

Just under 40% of 50 eligible learners completed the training and the first survey, while nearly 75% of those completed a follow-up survey. About 1 in 5 (*n* = 13, or 22%) of 60 eligible faculty completed the e-modules and survey, most of whom (*n* = 10) were from palliative care. Those learners completed both e-modules in time ranging from 21 to 60 minutes. Both learner and faculty participants rated the e-module highly: 100% of learners and all but one faculty agreed or strongly agreed that the e-module content met the learning objectives; 100% of faculty and all but one learner felt the content was appropriate; only three learners, all Family Medicine residents, did not agree or strongly agree that they would recommend the module to a colleague. All faculty agreed that they would be interested to use the module with learners. Moreover, the resident learners felt more confident in their ability to “empathically support” their patients (79%) and to lead serious illness conversations (90%). Eight of the learners who completed the follow-up survey reported revisiting the modules, and, more importantly, 86% and 79% of learners, respectively, reported using the communication skills and the structured conversation guide.

These results suggest a promising avenue for preparing clinicians to more effectively meet the needs of a growing proportion of patients, especially, as participants noted, as a supplement to didactic and bedside teaching. It also adds to a growing literature about educational modalities and outcomes in serious illness communication.^8^ I’m particularly encouraged that some residents went back to the modules more than once. My own experience suggests that medical students in clinical years and residents are hungry for content around serious illness communication. It meets a need that they feel acutely, based on the pressures of completing high-stakes communication tasks that their supervisors are often not themselves well trained to demonstrate. Given this, it would be important to understand why over half of the eligible learners did not complete the training. It may just be that residents feel busy and were not required to do it. If there were other barriers, however, it would be good to know.

Questions unanswered by this study are those with which many serious illness communication educators have long wrestled. For example, what happens when learners are trained to do one thing and enter a practice environment in which everybody else is doing something different? Serious illness communication skills are neither widely taught nor utilized by nonpalliative care faculty in medical schools and academic medical centers. This is not a critique but a sad reality that leaves patients and caregivers at risk of feeling treated-but-not-cared-for by clinicians. That only a small fraction of family medicine faculty completed the e-modules may suggest that they overestimate their own skills in this area or feel that it is not a priority. Or perhaps they feel they do not have the time, which is also a problem. This tracks with my and others experience across multiple institutions, in which systems need a combination of incentives and mandates to get clinicians to participate.

The biggest question of all is how we change institutional culture to recognize serious illness communication skills as a fundamental part of how we provide the highest quality health care. Part of this is increasing the recognition that these “soft skills” are the hardest skills in medicine to master. More than innovation in education modalities, we need teams focused on doing the hard work of implementation: of lobbying and cultivating leaders, of crafting messages and identifying incentives to gain clinician buy-in, and of developing measurement approaches to ensure that clinician confidence (a low bar to meet in studies of this kind) results in clinician behavior change.

As an institutional leader trying to assemble the resources, expertise, and commitment to change the culture of serious illness care, I recognize e-learning as a potentially useful piece of the puzzle. I certainly hope to see results of a larger trial of this approach with more resident and faculty participants. It will be most useful if that study is embedded in a larger implementation study that gives us more insights into how this and other pieces fit together. The resulting picture will not just change resident education; it will change medicine.

## References

[1] Greer JA, Applebaum AJ, Jacobsen JC, et al. Understanding and addressing the role of coping in palliative care for patients with advanced cancer. J Clin Oncol 2020;38(9):915–925. 32023161 10.1200/JCO.19.00013 PMC7082158

[2] Jackson VA, Emanuel L. Navigating and communicating about serious illness and end of life. N Engl J Med 2024;390(1):63–69. 38118003 10.1056/NEJMcp2304436

[3] Jacobsen J, Jackson V, Dahlin C, et al. Components of early outpatient palliative care consultation in patients with metastatic nonsmall cell lung cancer. J Palliat Med 2011;14(4):459–464; doi: 10.1089/jpm.2010.0382 21417739

[4] Van Dael J, Reader TW, Gillespie A, et al. Learning from complaints in healthcare: A realist review of academic literature, policy evidence and front-line insights. BMJ Qual Saf 2020;29(8):684–695. 10.1136/bmjqs-2019-009704 PMC7398301 32019824

[5] Arnold RM, Back AL, Baile WF, et al. The oncotalk/vitaltalk model. Oxford textbook of communication in oncology and palliative care. Published online 2017:363–368.

[6] Paladino J, Kilpatrick L, O’Connor N, et al. Training Clinicians in Serious Illness Communication Using a Structured Guide: Evaluation of a Training Program in Three Health Systems. J Palliat Med 2020;23(3):337–345; doi: 10.1089/jpm.2019.0334 31503520

[7] Kumar P, Paladino J, Gabriel PE, et al. The Serious Illness Care Program: Implementing a Key Element of High-Quality Oncology Care. NEJM Catalyst 2023;4(2):CAT.22.0309; doi: 10.1056/CAT.22.0309

[8] Lavecchia M, Myers J, Bainbridge D, et al. Education modalities for serious illness communication training: A scoping review on the impact on clinician behavior and patient outcomes. Palliat Med 2024;38(2):170–183; doi: 10.1177/02692163231186180 37424275 PMC10865772

